# Oral Health of Children and Adolescents with Diabetes Mellitus

**DOI:** 10.3390/jcm13226742

**Published:** 2024-11-09

**Authors:** Pauline Schädlich, Judit Symmank, Axel Dost, Collin Jacobs, Yvonne Wagner

**Affiliations:** 1 Center for Dental, Oral and Maxillofacial Medicine, Section Preventive and Pediatric Dentistry, University Hospital Jena, 07743 Jena, Germany; pauline.schaedlich@med.uni-jena.de; 2 Center for Dental, Oral and Maxillofacial Medicine, Department for Orthodontics, University Hospital Jena, 07743 Jena, Germany; judit.symmank@med.uni-jena.de (J.S.) collin.jacobs@med.uni-jena.de (C.J.); 3 Clinic for Pediatric and Adolescent Medicine, Section Diabetology, University Hospital Jena, 07747 Jena, Germany; axel.dost@med.uni-jena.de; 4 Dental Training Center Stuttgart, 70174 Stuttgart, Germany

**Keywords:** caries, diabetes, periodontitis, children, dentistry, ELISA, oral health

## Abstract

**Aim:** To examine the oral health of children and adolescents with and without diabetes mellitus. **Background:** Diabetes mellitus is the most common metabolic disease in childhood and demonstrates an increasing incidence. Many children live with gingivitis as a precursor to periodontitis. If left untreated, it can cause the development of periodontitis. The links between periodontitis and diabetes mellitus are known but have been little studied in the age group of children and adolescents. **Materials and Methods:** Clinical examination and collection of sulcus fluid from participants aged 5 to 21 years was performed. The following data were collected: demographic variables, caries prevalence, DMF-T, VPI, PUFA, salivary flow rate, HbA1c, PSI, and the concentration of IL-1β, IL-6, MMP-8, and TNF-α. **Results:** Patients with diabetes mellitus showed a significantly lower salivary flow rate with higher concentrations of MMP-8 and IL-1β. The data indicate that at this age, regular visits to the dentist are of great importance for the promotion of oral health in children and adolescents regardless of diabetes and that patients with diabetes mellitus in particular benefit from prevention, as they belong to the periodontitis risk group. **Conclusions**: Patients with low salivary flow rates and increased inflammatory mediators are high-risk patients for whom dental preventive measures play a major role.

## 1. Introduction

Diabetes mellitus is the most common chronic metabolic disease in children and adolescents, with increasing prevalence (1.52 million persons under 20) and incidence (149,500 new cases per year) worldwide [1]. The common feature of all types of diabetes is chronic hyperglycemia [2]. Type 1 diabetes mellitus is based on the destruction of the beta cells of the pancreas, resulting in an absolute insulin deficiency. Type 2 involves a reduced insulin effect, which is caused by peripheral insulin resistance [3]. The insulin receptor density decreases, which means that body cells absorb less glucose. The resulting high blood glucose concentration leads to insulin hypersecretion, which is why the resistance is intensified when insulin secretion decreases. A progressive reduction in the function of the beta cells or a disorder of glucose-dependent insulin secretion develops [4]. With around 175 compared to 3100 new cases of type 1 diabetes in Germany each year, the incidence of type 2 diabetes in children and adolescents is therefore significantly lower [5]. Specific types of diabetes due to other causes or type 3 diabetes is a group of diseases that have hyperglycemia as the main symptom but cannot be caused by other types of diabetes [6]. Gestational diabetes is present when glucose tolerance disorder is first diagnosed during pregnancy [7].

Hypoglycemia is the most common acute complication of diabetes mellitus [8]. Mild hypoglycemia can be treated by the patient by ingesting rapidly usable carbohydrates. In contrast, patients with severe hypoglycemia require outside help, as neurological symptoms can occur [9]. Hyperglycemia can be developed in unrecognized or inadequately treated diabetes mellitus. The development of microvascular and macrovascular diseases is considered a long-term risk. These include retino-, neuro-, and nephropathy as well as arteriosclerosis. If diseases such as nephropathy or retinopathy already occur in childhood and adolescence, this indicates a more severe course in the development of microvascular and macrovascular complications [8,10,11,12]. In addition, the occurrence of these diseases reduces life expectancy [13].

Reviews show that people with diabetes mellitus often have limited knowledge of oral health and inadequate oral hygiene behavior with a lower utilization of dental services [14]. In particular, patients with uncontrolled diabetes mellitus appear to be at increased risk of developing oral health problems [15,16,17,18,19,20]. Diabetes mellitus, periodontitis, and tooth decay are common diseases and share behavioral risk factors such as an unhealthy diet and lack of exercise [21,22].

Caries is caused by biofilm-related demineralization of the teeth and affects 43.6% of 6- to 7-year-olds in Germany [23]. This disease is preventable by reducing the frequency of sugar intake and maintaining adequate oral hygiene. According to the WHO, there is a causal link between high sugar consumption, diabetes mellitus, obesity, and dental caries [24]. However, the data on caries prevalence and caries experience in children with and without diabetes are limited and inconsistent [25,26,27,28,29,30,31,32]. More frequent meal intake, higher sugar concentration in saliva, and lower saliva flow rate are cited as causes for the differences in oral health [14,33,34,35,36]. Furthermore, poor oral hygiene and poorly controlled diabetes mellitus lead to a drop in the pH value and a reduction in the cleansing function of saliva [32]. Children and adolescents with diabetes mellitus appear to have an increased prevalence of xerostomia and a reduced salivary flow rate compared to control subjects [37]. Individuals with a low unstimulated salivary flow rate are more at risk of developing carious lesions due to the loss of the protective properties of saliva [38] and have poorer oral health with an increased number of decayed and filled teeth compared to patients with a physiological salivary flow rate [39]. At the same time, salivary flow rate correlates with diabetes control: the worse the metabolic control, the more frequently xerostomia and hyposalivation occur [39].

Periodontitis is a multifactorial disease that causes progressive destruction of the periodontium [40]. The bidirectional relationship between diabetes mellitus and periodontitis is considered certain, although the underlying mechanisms have not yet been conclusively clarified [15]. People with diabetes mellitus develop periodontitis at a younger age than healthy people [41]. The disease manifests itself as early as childhood [42] or adolescence [43]. The pathomechanisms have primarily been investigated in adults, but a similar pathogenesis is suspected in children [44]. As a systemic infection, periodontitis triggers increased tissue resistance to insulin [45]. At the same time, the onset and progression of periodontitis are influenced by microangiopathy, impaired immune system, reduced resistance to infection, altered oral microbiome, and dysfunction of collagen metabolism [46].

The null hypotheses were:

1. Children and adolescents with diabetes mellitus do not have poorer dental health than their healthy peers.
2. Diabetics have a higher salivary flow rate than healthy people.
3. Oral health-related quality of life does not differ between the study groups.
4. There are no differences in the concentrations of inflammatory parameters in the sulcus fluid between the study groups.

## 2. Materials and Methods

In this case-control study, the oral health of children and adolescents with and without diabetes mellitus was compared. People were recruited for the study during consultation hours at the Section for Preventive Dentistry and Pediatric Dentistry of the Department for Orthodontics and the Section for Diabetology of the Clinic for Pediatric and Adolescent Medicine at Jena University Hospital. Children and adolescents with and without diabetes mellitus were included.

### 2.1. Inclusion and Exclusion Criteria

Children and adolescents from the age of 5 to the age of 21 with diabetes mellitus and healthy children and adolescents were included. Children and adolescents in the diabetes group were included in the study regardless of their diabetes type. Since the influence of chronic hyperglycemia and long-term metabolic control on oral health is to be investigated, the cause or type of diabetes can be disregarded in this study. The following were excluded: healthy children and adolescents with other general medical conditions such as metabolic, cardiovascular, or tumor diseases; healthy children and adolescents who regularly take medication; children and adolescents who have taken antibiotics in the three months before the start of the study; adolescents who smoke (nicotine consumption); children and adolescents with fixed orthodontic appliances; missing or unsigned consent form. If the participants were minors, the informed consents were signed by the parents or guardians of the children.

### 2.2. Clinical Examination

The children and adolescents were examined according to WHO standards without taking X-ray images, using a WHO probe, dental forceps, and mouth mirror under dental lighting or an inspection lamp [47]. The teeth were dried using gauze swabs. The findings were recorded anonymously and coded on a documentation sheet. The caries prevalence and experience was recorded according to the WHO standard using the dmf-t/DMF-T index at dentin caries level [47]. In addition, initial carious lesions at enamel caries level were included in the findings and the index was extended to the tooth surface-related evaluation (“surface”) (dmf-s-/DMF-S-; dmfi-s/DMFI-S-Index). According to the Basic methods for oral health surveys (WHO), the dmf-t/DMF-T index was used with addition of initial caries lesions.

The pufa/PUFA index [48] was used to assess odontogenic infections as a result of untreated caries and the Visible Plaque Index [49] was used to classify the oral hygiene status.

The amount of saliva was determined using the saliva flow rate. Samples were collected in the morning or afternoon at least 2 h after eating, drinking, smoking, or tooth brushing [50]. The patient allowed saliva to drip into a scaled glass cup over a period of 5 min. After the time had elapsed, all the saliva in the mouth was spat into the funnel and the flow rate was read off the scaled tube. From this, the saliva flow rate per minute was calculated.

Sulcus fluid was collected by the examiners inserting paper strips into the sulcus of teeth 11, 12, 21, and 22.

A standardized evidence-based questionnaire (Oral Health Impact Profile OHIP-14) was used to determine oral health-related quality of life. If the study participants were too young, their parents or guardians helped to fill out the forms. 

The diabetes parameters (HbA1c, CPR, albumin content in urine, BMI) were collected as part of the routine examinations in the diabetes consultation. The children and adolescents were examined by two calibrated dentists. WHO-compliant calibration training [47] was carried out in advance by an epidemiologically experienced study and test dentist.

### 2.3. Sulcus Fluid Sample Preparation and ELISA

Frozen paper strips with sulcus fluid samples were incubated with 100 µL 1X Phosphate Buffered Saline (1X PBS) in Eppendorf tubes for 1 h at 4 °C to resolve proteins. Strips were clipped into the lid of the tube and centrifuged for 5 min at 1000× *g* at 4 °C [51]. For each sample, protein amount was measured with a BCA Protein-Assay-Kit (Thermo Fisher Scientific, Carlsbad, CA, USA) according to the manufacture’s guidelines. Sulcus fluid samples were analyzed for IL-1β, IL-6, MMP-8, and TNF-α using appropriate enzyme-linked immunosorbent assays (ELISA) according to the manufacturer’s guidelines. All samples were measured as technical duplicates. For each sample, measured cytokine concentration was correlated to the protein amount.

### 2.4. Statistics

The case number planning for this study was carried out by the Institute of Medical Statistics, Informatics and Data Science, Friedrich Schiller University Jena. The basis for the case number calculation were the periodontological aspects with the comparison of the inflammation parameters. In a pilot study, the amount of the inflammatory parameter interleukin 1-β was measured in sulcus fluid (mean value in patients without diabetes mellitus 0.658 ± 0.218 pg/mL vs. 1.365 ± 0.319 pg/mL in patients with diabetes mellitus). Based on this, the number of cases was planned at a power of 80% and a significance level of 5%, resulting in a number of 49 cases per group. 

Statistical analyses were performed with Graph Pad Prism 9 (https://www.graphpad.com; version number: 10.1.2, accessed on 26 November 2021). All experiments were performed in technical duplicates. Student’s *t*-test, Fisher’s exact test, and chi-square test were used as statistical tests. Significance levels: * *p*-value < 0.05, ** *p*-value < 0.01, *** *p*-value < 0.001.

## 3. Results

### 3.1. Population

Patient recruitment and examination took place from 11 December 2019 to 25 August 2020 at the Children’s Hospital of the University Hospital Jena and was made more difficult due to the corona pandemic, as children and adolescents with diabetes mellitus represent a risk group. The total population comprised 92 children and adolescents, 54 of whom had diabetes. The average age was 11.8 ± 4.1 years and differed significantly between the groups. Overall, 48.9% of participants were female, compared to 52.6% in the control group and 46.3% in the diabetes group.

### 3.2. Diabetes Mellitus Data

Diabetes control was assessed based on the HbA1c value (Table 1).

There was no significant difference between the genders. There was no significance for the relationship between BMI and diabetes control (Table 2).

Urine albumin levels correlated positively with diabetes control according to HbA1c levels. The annual diabetes program could not be carried out routinely during the corona pandemic. As a result, only isolated CRP values and 24-h blood pressure profiles were collected, which could not be statistically evaluated.

### 3.3. Caries Data

The caries prevalence of healthy individuals was 57.9% (CI 41.5–74.3%) and that of diabetics 42.6% (CI 29.0–56.2%) and did not differ significantly.

Deciduous teeth of children with diabetes mellitus showed a significantly lower caries experience than those without metabolic disease (Table 3). The diabetes setting correlated significantly and moderately negatively with the dmf-t/dmfi-t/dmf-s/dmfi-s.

There was no difference in caries experience in permanent dentition (Table 4). There was no significance for the weak positive correlation between caries experience and diabetes control according to the HbA1c value.

Among all study participants, only one participant with diabetes mellitus showed caries-related ulceration.

At 90.5% (CI 82.7–98.2%), patients without diabetes mellitus did not show a significantly higher degree of restoration than patients with diabetes mellitus (81.4% (CI 71.7–91.2%)). The same applies to the restoration index (control group 90.2% (CI 82.2–98.1%) vs. diabetes group 81.4% (CI 71.7–91.2%)).

Healthy subjects had an average Visible Plaque Index of 48.2% (CI 37.3–59.1%). At 63.0% (CI 53.9–72.1%), patients with diabetes mellitus achieved a significantly higher value. Diabetes control, according to the HbA1c value, had no influence on the VPI.

**Figure 1:**
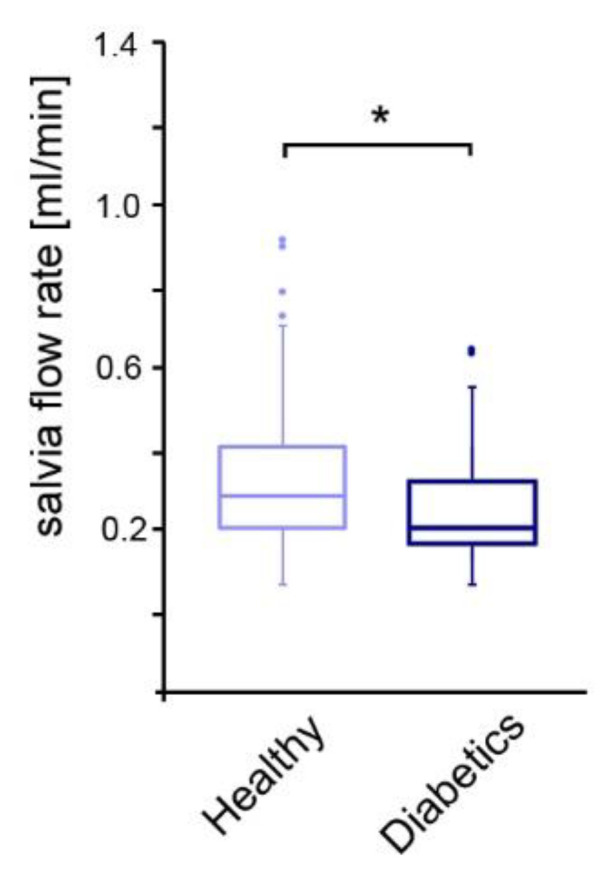
Comparison of salivary flow rate among the study groups. * *p*-value < 0.05, dots: outliers.

Metabolically healthy patients had a significantly higher salivary flow rate of 0.29 ± 0.22 mL/min compared to children and adolescents with diabetes mellitus with 0.19 ± 0.15 mL/min (Figure 1). Diabetes control, according to the HbA1c value, showed no detectable influence on the salivary flow rate.

### 3.4. Oral Health-Related Quality of Life

According to the total values of the Oral Health Impact Profile (OHIP-G 14), the study groups had a significant difference in oral health-related quality of life. Participants without diabetes mellitus had a higher mean sum score (4.19 ± 5.13) than participants with diabetes mellitus (2.04 ± 2.94).

### 3.5. Diabetic Patients Showed Increased IL-1β and MMP-8 Levels in Sulcus Fluids

**Figure 2:**
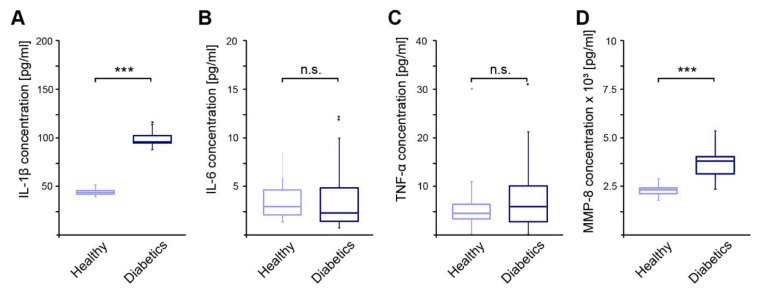
Concentrations of pro-inflammatory cytokines measured in sulcus fluids of diabetic patients in relation to healthy controls. (**A**)—Concentration of IL-1β; (**B**)—Concentration of IL-6; (**C**)—Concentration of TNF-α; (**D**)—Concentration of MMP-8; ***—*p*-value < 0.001; n.s.—not significant.

Persons with diabetes mellitus develop a chronic low-grade inflammation, which should be detected in the sulcus fluid. Concentrations of pro-inflammatory cytokines IL-1β, IL-6, TNF-α, and MMP-8 were measured in sulcus fluids of diabetic patients. While IL-6 and TNF-α levels were comparable to the healthy controls, significantly increased concentrations of IL-1β and MMP-8 were detected in the group of diabetic participants (Figure 2A–D).

## 4. Discussion

This study analyzed whether children and adolescents with diabetes mellitus have poorer dental health, with an increased caries prevalence and greater caries experience, compared to a healthy control group. Children and adolescents with diabetes mellitus were diagnosed with a lower caries prevalence compared to those without. In the literature, the prevalence of caries in minors with diabetes mellitus varies between 30% and 100%, depending on the study [54,55,56,57,58,59,60,61,62]. Contrary to the international state of knowledge, no correlation between metabolic control and caries prevalence could be established. In a systematic review and meta-analysis, Wang et al. investigated the prevalence of caries in children and adolescents with type 1 diabetes and found that patients with diabetes mellitus who were well controlled had a significantly lower prevalence of caries compared to those with poor control [63].

There were differences between the study groups in the caries experience of the primary dentition. Healthy people had significantly more caries than people with metabolic disease (1.3 vs. 0.5 dmf-t). The international literature is inconsistent. The majority of studies show that people without diabetes mellitus have less caries experience than those with diabetes mellitus [28,64,65,66,67], although the results are usually not significant. Some authors document a higher caries experience of the healthy study participants, also without significance [33]. The caries experience of permanent dentition showed no significant differences between patients with and without diabetes mellitus. Other studies also came to this conclusion [28,43,66,68].

Neither the degree of restoration nor the restoration index showed significant differences between the study groups.

Due to the small number, no significance could be determined for the PUFA index. International comparative values differ greatly because of the geographical location, caries experience, and level of education [69].

In the survey presented, patients in the diabetes group had a significantly higher VPI than those in the comparison group. VPI can be used to estimate the caries risk, as it represents a risk factor for caries development [70]. The documented discrepancy between the amount of plaque and caries experience can be explained by the examination modality. Healthy individuals came to the Center for Dental, Oral and Maxillofacial Medicine for a scheduled dental appointment, while participants with diabetes mellitus were only informed about the possibility of participating in the study during their pediatric examination. It can therefore be assumed that the children in the healthy group brushed their teeth beforehand with the knowledge of an upcoming visit to the dentist, whereas this was not performed in the group of children with diabetes.

Furthermore, the null hypothesis that diabetics have a higher salivary flow rate was disproved. People without diabetes mellitus showed significantly higher unstimulated salivary flow rates compared to those with diabetes. Numerous studies have also come to this conclusion [67,71,72]. Due to the different studies (age group, time of investigation, circadian rhythm, etc.) and the limited data available, the specific values are only comparable to a limited extent.

Consistent with oral health, participants without diabetes mellitus had a significantly higher OHIP-G sum score compared to those with diabetes mellitus. Although there were no significant differences between the groups for individual questions, which corresponds to the hypothesis, almost significant differences were found for questions on oral functionality. The control group was significantly younger, and the healthy participants were more likely to be in the second mixed dentition phase. It is possible that the change of teeth also impaired oral health-related quality of life.

The outbreak of the COVID-19 pandemic and the patient group of the Section of Preventive Dentistry and Pediatric Dentistry, which does not correspond to the epidemiological mean, had a limiting effect on the study.

It is well established that diabetes is a risk factor for the development of periodontitis. However, the exact pathomechanism is still the subject of intensive research. Priority has been given to adults, although a similar mechanism is suspected in children and adolescents.

As pro-inflammatory markers in the sulcus fluid, the cytokines IL-1b and IL-6 can be used to assess the inflammatory state of the periodontium [73].

The increased IL-1b levels in diabetics indicate an increased inflammatory state compared to the healthy control group. This corresponds to the current state of knowledge, and it has also been shown that there is a correlation between glycemic control and IL-1b concentration. Furthermore, younger diabetics appear to be increasingly affected by high IL-1b levels [74], which could be related to the cytokine storm associated with the first stage of type 1 diabetes [75].

IL-6 is causally involved in the development of insulin resistance in type 2 diabetes mellitus [76,77]. Since the patients in this study are primarily suffering from type 1 diabetes, it is therefore unlikely that any differences between healthy individuals and diabetics could be detected.

TNF-α stimulates IL-1b and IL-6 [78] and increases insulin resistance by phosphorylating insulin receptors. In this study, no difference in TNF-α concentrations was found between the study groups. Dos Santos Haber et al. report a change in the cytokine profile; over the course of the diabetes diagnosis, the TNF-α level increases [79]. It is possible that the duration of the disease in the population studied was still too short for the TNF-α concentration to differ from that of healthy individuals.

MMP8 has been established as a classic marker for periodontal inflammation. Diabetics exhibited elevated concentrations, but only the combination with clinical parameters and periodontal pathogens makes it possible to differentiate between stable and acute courses [80,81,82]. Due to the function of MMP8 in tissue degradation, an increased concentration represents a risk factor for the development of periodontitis.

### Limitations

The regionality and the clientele of the Section of Preventive Dentistry and Pediatric Dentistry had a limiting effect on this study. Due to its function as a maximum care provider, patients with a low social status and limited financial resources are treated. Despite a general decline in caries, groups from a low social milieu are increasingly at risk of developing caries [83]. To obtain more representative results, patients in private practices or in kindergartens and schools should be examined. In addition, the start of the study occurring shortly before the start of the global coronavirus pandemic had a limiting effect on the study. Due to the increased risk for diabetics, fewer medical check-ups took place, resulting in a small number of cases. The documented discrepancy between the amount of plaque and caries experience can be explained by the examination modality. Healthy individuals came to the Center for Dental, Oral and Maxillofacial Medicine for a scheduled dental appointment, while participants with diabetes mellitus were only informed about the possibility of participating in the study during their pediatric examination. It can therefore be assumed that the children in the healthy group brushed their teeth beforehand with the knowledge of an upcoming visit to the dentist, whereas this was not performed in the group of children with diabetes.

## 5. Conclusions

Finally, it can be stated that patients with low salivary flow rates and increased inflammatory mediators are high-risk patients for whom dental preventive measures play a major role.

## Figures and Tables

**Table 1:** Diabetes control based on HbA1c value [52].

HbA1c Value	Type of Control	*n*	%
**<7.5**	good	29	56.9
**7.5–9.0**	moderate	16	31.4
**>9.0**	poor	6	11.8

**Table 2:** BMI distribution [53] after diabetes control.

**Type of Control**		**Under Weight**	**Normal Weight**	**Over Weight**	**Obesity**	**Extreme Obesity**
	good	1 (3.4%)	17 (58.6%)	5 (17.2%)	3 (10.3%)	3 (10.3%)
	moderate	1 (6.3%)	12 (75.0%)	1 (6.3%)	1 (6.3%)	1 (6.3%)
	bad	0 (0.0%)	4 (66.7%)	1 (16.7%)	1 (16.7%)	0 (0.0%)
**all**		2 (3.9%)	33 (64.7%)	7 (13.7%)	5 (9.8%)	4 (7.8%)

**Table 3:** Caries experience in first dentition.

Index	All	Patients Without Diabetes	Patients with Diabetes	*p*-Value
dmf-t	0.8 ± 1.6	1.3 ± 1.9	0.5 ± 1.2	0.008
dmfi-t	1.1 ± 2.2	1.9 ± 2.6	0.6 ± 1.7	<0.001
dmf-t	1.7 ± 3.7	2.7 ± 4.2	1.0 ± 2.9	0.001
dmfi-t	2.1 ± 4.4	3.5 ± 5.3	1.2 ± 3.3	0.001

**Table 4:** Caries experience in second dentition.

Index	All	Patients Without Diabetes	Patients with Diabetes	*p*-Value
DMF-T	1.0 ± 2.7	1.2 ± 3.2	0.9 ± 2.3	0.365
DMFI-T	1.8 ± 3.4	1.7 ± 3.4	1.8 ± 3.4	0.889
DMF-S	1.9 ± 5.6	2.6 ± 7.8	1.4 ± 3.4	0.300
DMFI-S	2.6 ± 6.1	3.2 ± 7.9	2.2 ± 4.4	0.747

## Data Availability

The raw data supporting the conclusions of this article will be made available by the authors on request.
